# Impact of Paranasal Sinus Invasion on Oncologic and Dosimetric Outcomes in Nasopharyngeal Carcinoma Following Intensity-Modulated Radiation Therapy—Implications for Risk Stratification and Planning Optimization

**DOI:** 10.3389/fonc.2020.00407

**Published:** 2020-04-15

**Authors:** Xin Zhou, Xiayun He, Fen Xue, Xiaomin Ou, Chaosu Hu

**Affiliations:** ^1^Department of Radiation Oncology, Fudan University Shanghai Cancer Center, Shanghai, China; ^2^Department of Oncology, Shanghai Medical College, Fudan University, Shanghai, China

**Keywords:** nasopharyngeal carcinoma, paranasal sinus invasion, intensity-modulated radiation therapy, radiation dosage, prognosis

## Abstract

**Purpose:** This study aims to investigate the prognostic value and dosimetric impact of paranasal sinus invasion (PSI) in patients with nasopharyngeal carcinoma (NPC), and further to explore the feasibility of an integrative prognostic model based on anatomic, volumetric, and dosimetric features.

**Methods:** Two hundred six patients with T3 NPC receiving intensity-modulated radiation therapy (IMRT) were retrospectively analyzed. Dosimetric parameters were calculated from dose–volume histograms. Primary gross tumor volume (GTV-P) and dosimetric parameters were categorized using optimal cutpoints determined by R. Local recurrence-free survival (LRFS) was estimated using Kaplan–Meier method. Independent risk factors for LRFS were identified through univariable and multivariable analyses by Cox proportional hazards models.

**Results:** The incidence of PSI was 10.7% (22/206). Patients with PSI had significantly inferior 5-year LRFS (77.3 vs. 93.8%, *P* = 0.006). IMRT plans for patients with PSI had larger dose heterogeneity, higher frequency of underdosing, and higher maximum dose to optic structures. When categorized by optimal cutpoints, GTV-P > 38.67 cm^3^ (5-year LRFS, 84.8 vs. 97.4%, *P* = 0.008), and V66.88 < 89.87% (5-year LRFS, 67.1 vs. 94.5%, *P* < 0.001) were associated with significantly worse local outcome. Multivariable analyses showed that PSI, GTV-P > 38.67 cm^3^, and V66.88 < 89.87% were independent risk factors for local relapse, either in patients with or without concurrent chemotherapy. An integrative prognostic model was then established upon the cumulative score of risk factors. Subgroups with score of 0, 1–2, and 3 had distinctive local outcomes; the 5-year LRFS was 96.6, 84.7, and 58.3%, respectively (*P* < 0.001).

**Conclusions:** Paranasal sinus invasion jeopardized local control in T3 NPC patients due to large tumor burden and inadequate radiation dose in GTV-P. The presence of PSI, GTV-P, and radiation underdosing combined are critical for the risk stratification of local failure.

## Introduction

Nasopharyngeal carcinoma (NPC) is endemic in China and Southeast Asia, and radiotherapy is the mainstay treatment for non-metastatic patients. Over the past decades, intensity-modulated radiation therapy (IMRT) and chemotherapy have revolutionized the management of NPC (1) as well as the American Joint Committee on Cancer (AJCC) staging system (2). However, treatment difficulties remain in locally advanced NPC. With various patterns of tumor extension, locally advanced patients have different prognosis even under the same T category. Subclassification with high risk factors of local recurrence is needed to refine the current T staging. Previous studies have identified several subgroups with poorer local outcome in T4 NPC (3, 4), whereas for T3 disease, the data remained scarce.

Paranasal sinus invasion (PSI) occurs in ~30% NPC patients (5), and yet, it has been controversial in its prognostic value. In the Chinese 2008 staging system for NPC, PSI was classified as T4 (6), but it was retained within the T3 category in the 8th edition of AJCC staging system (7). Recently, cumulating data through MRI-guided diagnosis and IMRT-based treatment revealed that T3 NPC with PSI have similar 5-year local recurrence-free survival (LRFS) and overall survival (OS) with T4 disease (8, 9), suggesting the plausibility of further risk stratification within T3 classification.

It has been well-recognized that local control of NPC depends on radiation dosimetry (10, 11), which correlates closely to the extent of tumor invasion. For locally advanced NPC, the maximum tolerance dose of adjacent critical structures might increase the difficulty of IMRT planning, leading to underdosage in target volumes. Theoretically, invasion to paranasal sinuses, especially ethmoid sinus and upper sphenoid sinus, might complicate radiation dose distribution due to the anatomic proximity to optic nerves, chiasm, and temporal lobes. However, to date, the actual influence of PSI on IMRT dosimetry remains unclear, and how IMRT plan should be optimized in the existence of PSI needs to be explored.

In this study, based on comprehensive data from the clinic and radiation physics, we aimed to investigate the prognostic value of PSI status in T3 NPC patients, as well as its dosimetric impact on tumor and normal tissues. We also sought to establish a prognostic scoring system to stratify the risk of local recurrence, using both oncologic and dosimetric parameters.

## Methods and Materials

### Patient Population

Two hundred six consecutive non-metastatic T3 NPC patients receiving IMRT between June 2007 to December 2015 at our center were included in this retrospective study. All patients were histologically proven and restaged according to the 7th AJCC staging system (2010). Pretreatment evaluation included complete history and physical examination, hematology and biochemistry profiles, fiberoptic nasopharyngoscopy, MR scans of the head and neck, positron emission and computer tomography or substitutional bone scintigraphy, and chest and abdominal computed tomography. Data collection was conducted under the approval of our Institutional Review Board.

### Image Assessment

MR scans were performed using a 1.5-T scanner (Signa Excite HD, General Electric, Milwaukee, WI, United States) with a head-and-neck combined coil. The axial and sagittal T1-weighted fast spin-echo (FSE) images, axial T2-weighted FSE images, and contrast-enhanced T1-weighted fat suppression images in the axial and coronal planes were obtained. All images were reviewed independently by two radiologists experienced in head and neck cancers, and differences were resolved by consensus. The final conclusions were confirmed by the multidisciplinary team of NPC at our center before treatment. Details regarding the diagnostic criteria for paranasal sinus invasion have been published previously (12).

### Radiotherapy

The IMRT details at our center have been previously reported (13). Target volumes were delineated according to the International Commission on Radiation Units and Measurements (ICRU) Reports 50 and 62. Prescribed radiation dose was delivered in a schedule of five daily fraction per week, including 70.4 Gy in 32 fractions to the planning target volume (PTV) of primary gross tumor volume (GTV-P), 66 Gy to the PTV of nodal gross tumor volume (GTV-N), 60 Gy to the PTV of high-risk clinical target volume (CTV-1), and 54 Gy to the PTV of low-risk CTV (CTV-2). Normal structure constraints and compliance criteria were in agreement with Radiation Therapy Oncology Group protocol 0225 (14).

### Chemotherapy

All patients received at least one cycle of chemotherapy. Induction and adjuvant chemotherapy included TPF (docetaxel, 60 mg/m^2^ on day 1; cisplatin, 25 mg/m^2^/day on days 1–3; 5-fluorouracil, 500 mg/m^2^/day on day 1–5), TP (docetaxel, 60 mg/m^2^ on day 1; cisplatin, 25 mg/m^2^/day on days 1–3), PF (cisplatin, 25 mg/m^2^/day on days 1–3; 5-fluorouracil, 500 mg/m^2^/day on days 1–5), and GP (gemcitabine, 1,000 mg/m^2^ on days 1, 8; cisplatin, 25 mg/m^2^/day on days 1–3). Concurrent chemotherapy was administrated weekly (cisplatin, 30 mg/m^2^ on day 1).

### Follow-Up

After completion of treatment, patients were followed every 3 months during the first 2 years, every 6 months for the next 3 years, and then annually thereafter. The duration of follow-up was measured from the initiation of treatment to the last follow-up or death.

### Dosimetric Evaluation

Dose–volume parameters of IMRT plans were obtained from the treatment planning system, including maximum dose (Dmax), minimum dose (Dmin), dose that covered 98% (D98%), 50% (D50%), and 2% (D2%) of the target volume, and relative volumes that received a specific dose (Vds) (%). Dmax and dose to 1% of volume (D1%) for critical normal structures were also collected. Dose homogeneity within the target volume was evaluated using homogeneity index (HI), defined as the ratio of (D2–D98%)/D50% (15). Conformity of the plan was evaluated with Paddick conformity index (CI) based on the equation: CI = (TVPV/VPTV)/(VTV/TVPV), where VPTV is the volume of PTV, TVPV is the volume of VPTV covered by the prescribed isodose line, and VTV is the treated volume of the prescribed isodose line. CI value ranges from 0 to 1, and a value closer to 1 indicates better conformality (16).

### Statistical Analysis

All statistical analyses were performed using Statistical Product and Service Solutions 22.0 (SPSS Inc, Chicago, IL, United States) and R package (Version 3.5.2, http://www.R-project.org). Actuarial rates for LRFS, regional recurrence-free survival (RRFS), distant metastasis-free survival (DMFS), and OS were estimated using the Kaplan–Meier method. The “surv_cutpoint” function of Survminer package in R was used to implement the optimal cut-off point of continuous variables for LRFS, based on the maximally selected log-rank statistics. Univariable and multivariable survival analyses were conducted using the Cox proportional hazards model. Two-tailed *P* < 0.05 were considered statistically significant.

## Results

PSI occurred in 10.7% (22/206) of patients. Demographic and clinical characteristics of the patients are summarized in Table 1. With a median follow-up time of 63 months (range, 8–124 months), treatment failure was observed in 51 out of 206 patients (24.8%), of whom 9 (4.4%) developed local relapse only, 6 (2.9%) developed regional recurrence only, and 26 (12.6%) had isolated distant metastasis. Six patients (2.9%) developed both local and regional recurrence, one (0.49%) had both local and distant failure, and three (14.6%) had both regional and distant relapse. The overall 5-year LRFS, RRFS, DMFS, and OS was 91.9, 93.0, 85.8, and 88.3%, respectively.

**Table 1:** Patient characteristics.

**Characteristics**	**No. of patients (%)**
Age (years)	
≤ 49	103 (50.0)
>49	103 (50.0)
Gender	
Male	141 (68.4)
Female	65 (31.6)
KPS	
≤ 80	115 (55.8)
>80	91 (44.2)
Histology	
WHO I	0 (0)
WHO II/III	206 (100.0)
N classification (AJCC 7th)	
N0	22 (10.7)
N1	72 (35.0)
N2	82 (39.8)
N3	30 (14.6)
Clinical stage (AJCC 7th)	
III	176 (85.4)
IV	30 (14.6)
Chemotherapy	
Induction	192 (93.2)
Concurrent	32 (15.5)
Adjuvant	137 (66.5)

*KPS, Karnofsky performance status; AJCC, American Joint Committee on Cancer*.

### Effect of PSI on Dose Distribution

For the entire cohort in this study, Dmax, Dmin, and Dmean for GTV-P was 76.5, 64.0, and 72.5 Gy, respectively. Underdose of <66.88 Gy (95% prescribed dose) happened in 64.1% (132/206) of the patients. Dose–volume data based on the status of PSI are listed in Table 2. PSI tended to yield worse coverage of tumor volume (lower Dmean, Dmin, D98, and D95%) while generating more high-dose spots (higher Dmax and D2%). Underdosing happened more frequently in PSI-positive group (72.7 vs. 63.0%). Both PSI-positive and PSI-negative patients were well-covered with low dose (V51–V57); however, the percentage of GTV-P receiving higher dose (V60–V70.4) was significantly smaller in those with PSI. The comparisons of HI and CI indicated more prevalent dose heterogeneity in PTV for patients with PSI, although the plan conformity exhibited no difference between two subgroups.

**Table 2:** Comparison of target volume coverage based on paranasal sinus invasion.

**Parameters**	**Median (range)**		***P*-value**
	**PSI (–)**	**PSI (+)**	
GTV-P			
Volume (cm^3^)	30.4 (5.1–82.5)	45.5 (25.3–139.1)	0.003^*^
Dmin (Gy)	64.4 (53.1–71.7)	58.8 (50.0–70.6)	0.011^*^
Dmax (Gy)	76.4 (72.0–79.4)	77.0 (75.0–78.9)	0.002^*^
Dmean (Gy)	72.6 (68.6–75.1)	71.9 (68.0–74.2)	0.023^*^
D98% (Gy)	68.0 (56.0–72.6)	65.0 (51.4–72.2)	0.009^*^
D95% (Gy)	69.2 (61.0–73.0)	66.8 (52.4–72.7)	0.014^*^
D2% (Gy)	75.3 (71.2–78.1)	75.7 (73.8–77.4)	0.029^*^
V70.4 (%)	88.9 (6.3–100)	71.5 (37.0–100)	0.011^*^
V66.88 (%)	99.2 (78.2–100)	94.1 (54.7–100)	0.006^*^
V64 (%)	100 (89.2–100)	98.8 (76.8–100)	0.010^*^
V60 (%)	100 (96.0–100)	99.9 (80.6–100)	0.049^*^
V57 (%)	100 (97.2–100)	100 (86.8–100)	0.146
V54 (%)	100 (99.8–100)	100 (89.0–100)	0.257
V51 (%)	100 (100–100)	100 (98.4–100)	0.329
HI	0.15 (0.05–0.35)	0.20 (0.10–0.34)	0.002^*^
CI	0.86 (0.44–0.91)	0.83 (0.63–0.90)	0.527

*PSI, paranasal sinus invasion; GTV-P, gross volume of primary tumor; HI, homogeneity index; CI, conformity index*.

* *P < 0.05*.

Radiation exposure of critical organs are presented in Table 3. Ipsilateral optic nerves had higher Dmax and D1% (*P* < 0.01) than the contralateral ones, regardless of PSI status. However, no significant difference was observed between two sides of temporal lobes. Compared to PSI-negative patients, PSI-positive group had significantly higher Dmax and D1% in brain stem, optic chiasm, and both sides of optic nerves. No difference was observed in spinal cord, temporal lobes, or pituitary between the two groups.

**Table 3:** Dosimetric comparison of organs at risk based on paranasal sinus invasion.

**OARs**	**Parameters**	**Median (range)**		***P*-value**
		**PSI (–)**	**PSI (+)**	
Brain stem	Dmax	55.4 (52.4–59.1)	56.6 (53.3–60.2)	0.039^*^
	D1%	52.5 (46.8–56.1)	53.0 (49.7–56.1)	0.048^*^
Spinal cord	Dmax	43.6 (39.7–47.9)	44.3 (42.7–45.0)	0.176
	D1%	40.9 (36.1–43.7)	41.7 (39.7–43.4)	0.127
Chiasm	Dmax	55.1 (32.4–60.1)	57.3 (45.4–61.5)	0.000^*^
	D1%	54.1 (32.1–59.9)	56.7 (45.2–60.4)	0.024^*^
ipsi_OPN	Dmax	52.9 (29.5–58.8)	55.9 (46.6–60.3)	0.015^*^
	D1%	51.9 (28.6–58.3)	56.3 (46.4–59.7)	0.015^*^
contra_OPN	Dmax	51.6 (23.5–58.7)	55.9 (45.5–59.7)	0.000^*^
	D1%	50.6 (23.1–58.1)	55.3 (44.2–59.1)	0.000^*^
ipsi_TL	Dmax	65.3 (62.6–70.4)	65.2 (64.4–68.2)	0.892
	D1%	63.7 (58.2–68.4)	63.3 (61.7–64.8)	0.799
contra_TL	Dmax	64.8 (61.3–68.0)	65.0 (64.5–68.9)	0.145
	D1%	62.7 (55.9–66.5)	62.8 (61.6–66.8)	0.407
Pituitary	Dmax	59.7 (48.9–66.3)	57.9 (50.6–67.2)	0.273
	D1%	59.6 (48.4–66.3)	57.8 (50.5–67.1)	0.315

*OAR, organ at risk; PSI, paranasal sinus invasion; OPN, optic nerve; TL, temporal lobe; ipsi_, ipsilateral; contra_, contralateral*.

* *P < 0.05*.

### Impact of PSI on Local Control

**Figure 1:**
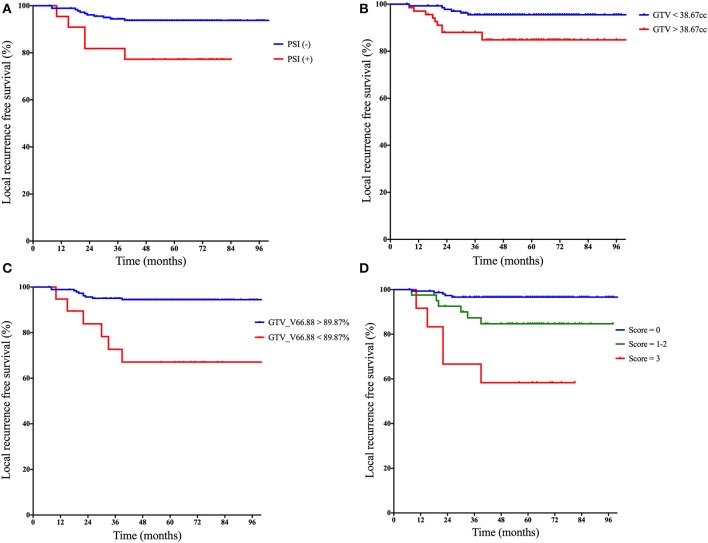
Kaplan–Meier curves of local recurrence-free survival based on: **(A)** the status of PSI, **(B)** categorical GTV, **(C)** categorical V66.88 for GTV-P, and **(D)** the prognostic scores derived from the quantification of following risk factors: (1) paranasal sinus invasion-positive, (2) GTV > 38.67 cm^3^, and (3) V66.88 for GTV <89.87%. PSI, paranasal sinus invasion; GTV, gross tumor volume; V66.88, target volume covered with dose over 66.88 Gy.

Within the follow-up period, 5 out of 22 (22.7%) patients with PSI and 11 out of 184 (6.0%) patients without PSI experienced local failure. The 5-year LRFS for PSI-positive and PSI-negative patients was 77.3 and 93.8%, respectively (log-rank test, *P* = 0.006) (Figure 1A).

### Impact of Primary Tumor Volume on Local Control

The median GTV-P in the whole series was 31.4 cm^3^ (range, 5.1–139.1 cm^3^). PSI-positive patients had significantly larger GTV-P than PSI-negative group (median, 45.5 vs. 30.4 cm^3^, *P* = 0.003). According to maximally selected log-rank statistics in R, the optimal cut point of GTV-P for predicting LRFS was 38.67 cm^3^. The estimated 5-year LRFS for patients with GTV-P > 38.67 cm^3^ was significantly lower than those with GTV-P ≤ 38.67 cm^3^ (84.8 vs. 95.5%; log-rank test, *P* = 0.008) (Figure 1B).

### Impact of Dose Coverage on Local Control

**Figure 2:**
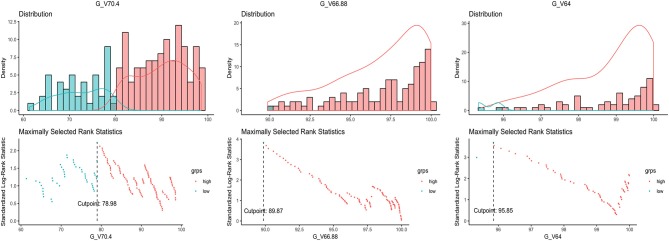
Distribution of the dosimetric parameters most significantly related to local recurrence and the maximally selected rank statistics for the optimal cutpoints.

Distribution pattern and maximally selected log-rank statistics of most significant dosimetric parameters for predicting LRFS are shown in Figure 2. Using the cutpoint of 89.87%, categorical V66.88 was identified as the best prognosticator for LRFS (standardized log-rank statistic = 3.83). V66.88 < 89.87% for GTV-*P* (>10.13% of GTV-P received <95% prescribed dose), which occurred in 5.4% (10/184) of PSI-negative and 40.9% (9/22) of PSI-positive patients, lead to a significantly lower 5-year LRFS in the whole population (67.1 vs. 94.5%; log-rank test, *P* < 0.001) (Figure 1C).

### A Prognostic Score-Based Risk Stratification for Local Relapse

In univariable analyses, Karnofsky performance status, PSI status, categorical GTV-P, and V66.88 strongly correlate with LRFS, while age showed only a marginal significance. After adjustment for potential confounders in a multivariable regression model, PSI, categorical GTV, and V66.88 remained predictive of local outcome (Table 4) (*P* < 0.05). No correlation was found between LRFS and chemotherapy or duration of radiotherapy. Considering the clinical significance of concurrent chemotherapy on improving local control, another multivariable regression analysis was also performed in the subgroup of patients receiving no concurrent chemotherapy (*n* = 174), finding similar results (Table 5).

**Table 4:** Univariable and multivariable Cox regression analyses for local recurrence-free survival.

**Variable**	**Univariable**		**Multivariable**	
	**HR (95% CI)**	***P*-value**	**HR (95%CI)**	***P*-value**
Age		0.056		
≤ 49	1 (reference)			
>49	3.02 (0.97–9.36)			
Gender		0.544		
Male	1 (reference)			
Female	0.70 (0.23–2.18)			
KPS		0.025^*^		
≤ 80	1 (reference)			
>80	0.28 (0.11–0.62)			
N classification (AJCC 7th)		0.661		
N0	1 (reference)			
N1	1.86 (0.31–7.14)			
N2	0.73 (0.13–3.98)			
N3	1.23 (0.25–5.90)			
Clinical stage (AJCC 7th)		0.177		
III	1 (reference)			
IV	2.00 (0.73–5.53)			
Paranasal sinus invasion		0.010^*^		0.047^*^
No	1 (reference)		1 (reference)	
Yes	3.53 (1.75–7.12)		2.69 (1.01-7.21)	
GTV-P > 38.67 cm^3^		0.013^*^		0.024^*^
No	1 (reference)		1 (reference)	
Yes	3.48 (1.31–9.25)		3.04 (1.19–7.78)	
V66.88 for GTV < 89.87%		<0.001^*^		0.009^*^
No	1 (reference)		1 (reference)	
Yes	5.57 (2.40–12.94)		3.82 (1.42–10.30)	
Neoadjuvant chemotherapy		0.904		
No	1 (reference)			
Yes	0.88 (0.12–6.48)			
Concurrent chemotherapy		0.725		
No	1 (reference)			
Yes	1.30 (0.30–5.43)			
Adjuvant chemotherapy		0.358		
No	1 (reference)			
Yes	1.59 (0.59–4.27)			
Duration of radiotherapy(d)		0.683		
≤ 46	1 (reference)			
>46	1.25 (0.44–3.59)			

*KPS, Karnofsky performance status; AJCC, American Joint Committee on Cancer; GTV-P, gross volume of primary tumor; HR, hazard ratio; CI, confidence interval*.

* *P < 0.05*.

**Table 5:** Univariable and multivariable Cox regression analyses for local recurrence-free survival in patients with no concurrent chemotherapy.

**Variable**	**Univariable**		**Multivariable**	
	**HR (95%CI)**	***P*-value**	**HR (95%CI)**	***P*-value**
Age		0.059		
≤ 49	1 (reference)			
>49	3.16 (0.96–9.93)			
Gender		0.370		
Male	1 (reference)			
Female	0.56 (0.16–2.00)			
KPS		0.027^*^		
≤ 80	1 (reference)			
>80	0.38 (0.13–0.75)			
N classification (AJCC 7th)		0.856		
N0	1 (reference)			
N1	1.34 (0.94–2.80)			
N2	0.78 (0.16–3.96)			
N3	0.79 (0.11–5.61)			
Clinical stage (AJCC 7th)		0.907		
III	1 (reference)			
IV	0.92 (0.21–3.03)			
Paranasal sinus invasion		0.007^*^		0.046^*^
No	1 (reference)		1 (reference)	
Yes	3.82 (1.49–9.79)		2.72 (1.05–6.97)	
GTV-P > 38.67 cm^3^		0.019^*^		0.021^*^
No	1 (reference)		1 (reference)	
Yes	3.41 (1.21–9.59)		3.76 (1.33–10.62)	
V66.88 for GTV < 89.87%		<0.001^*^		0.007^*^
No	1 (reference)		1 (reference)	
Yes	4.97 (2.40–14.18)		5.19 (1.63–16.52)	
Neoadjuvant chemotherapy		0.894		
No	1 (reference)			
Yes	1.15 (0.14–8.69)			
Adjuvant chemotherapy		0.364		
No	1 (reference)			
Yes	1.58 (0.59–4.24)			
Duration of radiotherapy (days)		0.688		
≤ 46	1 (reference)			
>46	1.24 (0.43–3.57)			

*KPS, Karnofsky performance status; AJCC, American Joint Committee on Cancer; GTV-P, gross volume of primary tumor; HR, hazard ratio; CI, confidence interval*.

* *P < 0.05*.

A prognostic scoring system was then established upon these three risk factors: (a) PSI positive, (b) GTV-P > 38.67 cm^3^, and (c) V66.88 < 89.87%. The score for each patient was calculated as the number of existing risk factors. One hundred twenty-five, 62, 10, and 9 patients were scored 0, 1, 2, and 3, respectively. In the subgroup with score = 1, 49 patients had GTV-P > 38.67 cm^3^ only, 6 had PSI only, and 7 had underdosing only; in the subgroup with score = 2, 3 patients had both GTV-P > 38.67 cm^3^ and underdosing, while 7 had both GTV-P > 38.67 cm^3^ and PSI. Based on this scoring system, the whole population was divided into three subgroups: (1) score = 0, (2) score = 1–2, and (3) score = 3. This classification yielded a well-stratified local relapse hazards, and the 5-year LRFS for each subgroup was 96.6, 84.7, and 58.3%, respectively (log-rank test, *P* < 0.001) (Figure 1D). Compared with the prognostic models with single-factor of PSI (c-index = 0.603), GTV (c-index = 0.649), and GTV_V66.88 (c-index = 0.641), the integrative model showed significantly improved predictive efficiency of LRFS (c-index = 0.738).

## Discussion

To our knowledge, this is the first study to incorporate dosimetric parameters with PSI for prognostication in NPC patients. Meanwhile, we demonstrated for the first time the dosimetric factors accounting for the prognostic value of PSI on local control.

PSI is not rare in NPC. Previously reported incidence of PSI ranged from 16.0% (17) to 42.7% (18) in locally advanced NPC. However, most of studies included both T3 and T4 patients. For T3 and T4 classification, the incidence of PSI was 11.0–28.0% and 45.2–57.6%, respectively. In our study, only T3 patients were analyzed. T4 patients were excluded since the prognostic value of PSI in this population is hard to isolate from more extensive lesions, e.g., intracranial involvement. As a result, we found a relative low incidence of PSI in this study (10.7%), which was quite similar to Zhang's report in T3 NPC (17).

The prognostic value of PSI has long been equivocal for NPC. Since the 5th edition of AJCC staging system, PSI has been retained in T3 category (19, 20). However, this classification was based mainly on CT imaging and conventional radiotherapy; with modern techniques of MRI and IMRT, NPC has undergone tremendous changes in patterns of failure, and the role of PSI in long-term survival should be re-evaluated. Following the latest 8th edition of AJCC staging, new evidence of PSI has emerged. In a 1,811-case series, Zhang et al. reported that T3 NPC with ethmoid or maxillary sinus invasion had a local recurrence rate comparable to T4 disease; thus, it should be upgraded to T4 (17). Similar results were found by Cao et al. (8). Another study showed that classification of PSI into T4 provided better distinction between T3 and T4 in all survival outcomes (18). Our study demonstrated the independent prognostic value of PSI after ruling out the confounding of T4 structures. Two hundred six T3 patients were divided by PSI into two groups with 5-year LRFS of 77.3 and 93.8%, which was similar to T2 and T4 disease, respectively, in our previous report (13). These data supported the value of PSI for subclassification in T3 NPC, as well as its potential for further optimization of AJCC T staging in future.

Primary tumor volume has been recognized as a complement of anatomy-based T staging in NPC for predicting LRFS. Multiple studies have shown that larger GTV-P correlates with worse local control after conventional radiotherapy (21–23). Using three-dimensional conformal techniques, Sze et al. also found that GTV-P ≥ 15 cm^3^ led to remarkably lower 3-year LRFS and OS (24). Similar effects remained in the era of IMRT. Recent IMRT studies revealed that MRI-derived GTV-P could serve as a direct indicator of tumor burden and an outstanding prognosticator of local recurrence, especially in locally advanced NPC (25–27). Pan et al. reported that GTV-P provided improved prognostic accuracy in addition to the 8th edition of AJCC staging system (28). In our study, GTV-P was found to be independently predictive of LRFS even within the same T3 category, suggesting its potential to further distinguish local outcome on the basis of current T staging. The optimal cutpoint for GTV-P was estimated to be 38.67 cm^3^, which was quite close to Feng's estimation (27). However, one should take caution when applying this cutoff GTV-P to other patients, since the optimal cutpoint could vary with different population structure. In fact, the cutoff GTV-P ranged widely in previous reports from 15 to 65.7 cm^3^ (29, 30), and a well-acknowledged cut point is still lacking. Further studies are warranted to determine the feasibility of a universal categorization scheme of GTV-P for prognosis in future.

The importance of GTV-P was believed to have biological and dosimetric fundaments. First, larger tumor might have more clonogenic tumor cells associated with relapse propensity (31). Increased tumor size could also induce hypoxia and radioresistance, requiring higher radical dose of radiation (32). More importantly, larger GTV-P tends to narrow the distance to critical organs, causing inadequacy of radiation dose in target volumes due to the limitation of normal tissue tolerance. According to Ng et al. most patients with T4 disease were underdosed with 66.5 Gy, leading to a remarkably impaired 5-year LRFS, DFS, and OS (10). Our previous data in locally advanced NPC found that, although with good responses after induction chemotherapy, T4 disease with lower Dmin of GTV-P correlated with worsened LRFS (33). For T3 NPC, tumor size is smaller, theoretically enabling better dose distribution in target volumes; therefore, it might be reasonable to adopt more stringent requirements for dose coverage of GTV in IMRT. However, the best dosimetric threshold in T3 NPC remains undefined so far. In our study, V66.88 of GTV-P was identified as the most predictive factor for local recurrence following IMRT, and the optimal cutoff value was 89.87%, suggesting that percentage of GTV-P receiving <66.88 Gy should not exceed 10.13%. These results hopefully provide evidence for plan evaluation in future, although more data will still be needed for further validation.

Our dosimetric investigation showed that PSI status was a major factor that impacts GTV dosimetry in T3 patients. PSI significantly jeopardized IMRT plans, causing higher heterogeneity and poorer GTV coverage. Underdose to over 10.13% of GTV occurred in over 40% of patients with PSI, compared to only 5.4% of those without. Meanwhile, the exposure dose of optic chiasm and both sides of optic nerves were remarkably higher in patients with PSI, mostly approaching the maximum tolerance dose. These results suggested that PSI, with its special anatomic location, narrowed down the therapeutic window of radiation mainly through the trade-off between visual pathway toxicity and tumor eradication. Similar condition was seen in definitive radiotherapy for primary paranasal sinus malignancies. Daly et al. reviewed their IMRT dosimetry in 36 patients with sinonasal malignancies and found that with median prescribed dose of 70 Gy to GTV, an average of 6.4% of GTV was underdosed, while the ipsilateral optic nerve received a median Dmax as high as 59.1 Gy (34). Another study in locally advanced paranasal sinus tumors showed that with dose limit of Dmax <60 Gy to bilateral optic nerves, 9/13 of the IMRT plans had over 5% of PTV receiving inadequate dose (lower than 95% of prescribed dose).

There are several prospects to address the treatment difficulties caused by PSI. First, deintensification of radiation after induction chemotherapy (IC) is potentially feasible to reduce toxicities. According to Yang et al. (35) and Zhao et al. (36), delineation of GTV on post-IC MRI while lowering prescription dose to shrink GTV significantly decreased radiation dose to organs at risk without damaging long-term survival. Niu et al. reported encouraging treatment outcome with reduced dose to optic nerves and chiasm by mid-course replanning during radiation following IC (37). Second, improved understandings of optic pathway tolerance to radiation will benefit clinical decisions in the dilemma between tumor control and vision protection. Although Dmax <54–55 Gy has been widely recommended for inverse IMRT planning, it is gradually accepted that optic pathway can safely tolerate up to 60 Gy and even higher (38). Therefore, in the case of PSI, much less conservative constraints for optic structures could be considered to yield higher dose coverage in GTV-P. In addition, proton therapy, with its superiority in conformality, allows for better sparing of normal tissues (39) and thus merits future investigation.

The present study established a prognostic score-based risk stratification system, combining both pretreatment tumor characteristics and treatment-related dosimetric factors. The high-risk subgroup in T3 patients with all three risk factors had a 5-year local recurrence rate of 41.7%, even exceeding that of T4 patients in previous reports. This stratification will benefit not only in better discriminating prognosis but also in guiding personalized treatment. For instance, for patients with both PSI and larger GTV, the focus should primarily be how to minimize the area of GTV receiving lower than 66.88 Gy during IMRT optimization. Meanwhile, intensified chemotherapy could be considered to offer a systemic effect of reducing tumor burden. On the contrary, PSI-negative T3 patients with smaller GTV and better dose distribution tend to have similar local outcome to early stage NPC. This low-risk group could be a potential candidate for treatment deintensification in future.

## Conclusion

This study demonstrated PSI as an independent predictor of local recurrence in T3 NPC patients. The prognostic scoring model based on pretreatment PSI status, GTV, and underdosed volume of GTV enables further risk stratification within T3 NPC. The results provide evidence to future refinement of AJCC staging, as well as treatment options. However, due to the retrospective nature, data bias was inevitable in this study. In addition, our sample size was relatively small. More data on long-term prognosis as well as treatment-related toxicities will be needed in the future before solid conclusions can be drawn. Moreover, as the study cohort consisted mostly of patients with no concurrent chemotherapy, our conclusions would pertain to those who refuse or are ineligible for concomitant chemotherapy.

## Data Availability Statement

The datasets generated for this study are available on request to the corresponding author.

## Ethics Statement

The studies involving human participants were reviewed and approved by Institutional Review Board, Fudan University Shanghai Cancer Center. The patients/participants provided their written informed consent to participate in this study. Written informed consent was obtained from the individual(s) for the publication of any potentially identifiable images or data included in this article.

## Author Contributions

XZ: data acquisition and manuscript drafting. FX: statistical analysis. XO: language modification. XH: conceptualization, design, manuscript review, and editing. CH: supervision of the study. All authors had read and approved the final manuscript.

### Conflict of Interest

The authors declare that the research was conducted in the absence of any commercial or financial relationships that could be construed as a potential conflict of interest.
